# Lipids at the Crossroad of α-Synuclein Function and Dysfunction: Biological and Pathological Implications

**DOI:** 10.3389/fncel.2019.00175

**Published:** 2019-05-01

**Authors:** Natalia P. Alza, Pablo A. Iglesias González, Melisa A. Conde, Romina M. Uranga, Gabriela A. Salvador

**Affiliations:** ^1^Instituto de Investigaciones Bioquímicas de Bahía Blanca, Consejo Nacional de Investigaciones Científicas y Técnicas, Universidad Nacional del Sur, Bahía Blanca, Argentina; ^2^Departamento de Química, Universidad Nacional del Sur, Bahía Blanca, Argentina; ^3^Departamento de Biología, Bioquímica y Farmacia, Universidad Nacional del Sur, Bahía Blanca, Argentina

**Keywords:** α–synuclein, lipids, lipid metabolism, lipid signal transduction, membrane lipids

## Abstract

Since its discovery, the study of the biological role of α-synuclein and its pathological implications has been the subject of increasing interest. The propensity to adopt different conformational states governing its aggregation and fibrillation makes this small 14-kDa cytosolic protein one of the main etiologic factors associated with degenerative disorders known as synucleinopathies. The structure, function, and toxicity of α-synuclein and the possibility of different therapeutic approaches to target the protein have been extensively investigated and reviewed. One intriguing characteristic of α-synuclein is the different ways in which it interacts with lipids. Though in-depth studies have been carried out in this field, the information they have produced is puzzling and the precise role of lipids in α-synuclein biology and pathology and *vice versa* is still largely unknown. Here we provide an overview and discussion of the main findings relating to α-synuclein/lipid interaction and its involvement in the modulation of lipid metabolism and signaling.

## Introduction

α-Synuclein is a cytosolic protein of 140 amino acids which was discovered in 1988 together with β- and γ-synucleins by Maroteaux et al. (1988). The name α-synuclein derives from the fact that it was originally described as being located in presynaptic endings and also as being associated with nuclear envelopes. In the central nervous system, α-synuclein is abundantly expressed in neurons of different brain areas such as the neocortex, hippocampus, SN, thalamus, and cerebellum (Iwai et al., 1995; Asi et al., 2014). α-Synuclein is also present in the peripheral nervous system, muscle, liver, heart, lungs, kidney, hematopoietic cells of the bone marrow, and circulating blood cells(Gardai et al., 2013; Burré et al., 2018; Mohamed Badawy et al., 2018). β-Synuclein is also located at presynaptic terminals in the central nervous system (Jakes et al., 1994; Wilhelm et al., 2014) while γ-synuclein is primarily expressed in the peripheral nervous system, and the ocular and adipose tissues (Buchman et al., 1998; Surguchov et al., 2001). It has been reported that γ-synuclein is overexpressed in different human tumors (Bruening et al., 2000; Guo et al., 2007; Hibi et al., 2009).

Though numerous studies have been carried out on the biology of α-synuclein, its physiological function remains a matter of debate. The search for a fuller understanding of this function is driven by the fact that it has been linked with several devastating diseases known as synucleinopathies, including neurodegenerative disorders such as: PD, Lewy body dementia, neurodegeneration with brain iron accumulation, Krabbe disease, dementia with LB, diffuse Lewy body disease, Lewy body variant of Alzheimer’s disease, among others (Burré, 2015; Burré et al., 2018).

Parkinson’s disease is the second most prevalent neurodegenerative age-associated disorder after Alzheimer’s disease and is mainly characterized by movement impairments such as resting tremor, bradykinesia, and rigidity. The loss of dopaminergic neurons in the SN has been identified as the cause of the typical motor disablement (Dunnett and Björklund, 1999). Current therapies focus on the reestablishment of the neurotransmitter dopamine for controlling motor symptoms, but specific treatments are still not available owing mainly to a lack of knowledge of the molecular mechanisms that trigger neuronal degeneration and death (Oertel and Schulz, 2016). Since diagnosis occurs when the loss of dopaminergic neurons is massive, a better understanding of the molecular events involved in the neurodegenerative process would undoubtedly help in the discovery of specific treatments.

Parkinson’s disease is a multifactorial neurodegenerative disorder whose etiopathogenesis is still largely unknown, though a common finding in patients’ brain is the abnormal accumulation, and aggregation of α-synuclein. Intracellular aggregates of α-synuclein, named LB, constitute the histological hallmark of the PD brain. LB are also the main histopathological findings in the above-mentioned synucleinopathies. The role of α-synuclein as one of the leading causes of dopaminergic cell death was described after the identification of the first missense mutations (A30P, E46K, and A53T) in the SNCA gene (Polymeropoulos et al., 1997; Krüger et al., 1998; Zarranz et al., 2004). The involvement of α-synuclein mutants in familial inherited PD has been confirmed by genome-wide association studies (Chang et al., 2017). In addition, SNCA duplication and triplication causing elevated levels of the protein are associated with early-onset PD (Singleton et al., 2003; Chartier-Harlin et al., 2004). Although the α-synuclein protein does not display any mutations in sporadic PD and LB dementia, its involvement in neuronal damage is widely recognized. Of the several mechanisms that could be responsible for the association of α-synuclein with PD pathogenesis, there is an overall consensus that its aggregation leading to the formation of oligomers is a central event related to neuronal dysfunction (Ingelsson, 2016; Bengoa-Vergniory et al., 2017). These α-synuclein oligomeric forms are considered the most toxic species, disrupting cellular homeostasis and triggering neuronal death. Moreover, α-synuclein can exert a deleterious effect by spreading from cell to cell and thus contributing to progressive neurodegeneration (Desplats et al., 2009). Identification of the factors promoting the toxic conversion of mutated or wild-type forms of α-synuclein is a topic of intense interest. Deeper insight into the physiological function and pathological features of α-synuclein would not only contribute toward a better understanding of the pathogenesis but also help to develop biomarkers for early disease detection and progression and to design specific disease-modifying therapies for synucleinopathies.

The α-synuclein protein is composed of three well-described regions that confer the biological and functional characteristics that implicate it in PD pathology. The N-terminal domain comprises residues 1–60 and bestows lipid binding properties on the protein, in particular, the ability to interact with membranes and lipid micelles. This distinctive feature of α-synuclein is explained by the presence of amphipathic repeats of 11 amino acids, predominantly with the highly conserved KTKEGV motif, similar to that present in apolipoprotein domains (Bussell and Eliezer, 2003). This structural characteristic is shared with β- and γ-synucleins (Clayton and George, 1998). The missense mutations A53T, A30P, H50Q, G51D, A53E, and E46K reside in the N-terminal domain of α-synuclein (Polymeropoulos et al., 1997; Krüger et al., 1998; Zarranz et al., 2004; Flagmeier et al., 2016). The central hydrophobic region (residues 61–95) called NAC (non-amyloid β component) is essential for the conformational change of α-synuclein from random coil to β-sheets, leading to aggregation, and fibrillation (El-Agnaf et al., 1998; Rodriguez et al., 2015). Giasson et al. (2001) demonstrated through different assays that amino acids 71–82 represent the specific sequence of the NAC core which is necessarily involved in α-synuclein fibrillation. Biophysical studies using methods like nuclear magnetic resonance, electron paramagnetic resonance, circular dichroism and transmission electron microscopy, with recombinant proteins of α-synuclein and synthetic fragments of NAC were the first and are still used to demonstrate the importance of this region in the fibrillation pathway (El-Agnaf et al., 1998; Giasson et al., 2001; Der-Sarkissiant et al., 2003; Tashiro et al., 2008; Waxman et al., 2009; Shaltiel-Karyo et al., 2010). The elimination of the NAC region or its targeting with antibodies showed to inhibit aggregation and toxicity in cell culture (Lynch et al., 2008; Luk et al., 2009). *In vivo* models were also used to evaluate the pathological effects of this region. For instance, the deletion of amino acids 71–82 in *Drosophila* prevents α-synuclein aggregation and neurotoxicity, thus testifying to the importance of this region in the pathogenicity of the protein (Periquet et al., 2007). Differences in the amino acid sequence of the NAC region have been attributed to be responsible for the non-fibrillation feature of β-synuclein (Uversky and Fink, 2002). The third region of α-synuclein, enriched in glutamate, aspartate, and proline residues, is the acidic C-terminal domain (residues 96–140). It constitutes the main site of post-translational modifications, such as phosphorylation and nitration, although other modifications (ubiquitination, glycation, and methionine oxidation) also occur in the N-terminal domain (Chen et al., 2019). These modifications alter α-synuclein structure and provoke changes in hydrophobicity and protein-protein and protein-lipid interactions (Burré et al., 2018). Oxidation, nitration and phosphorylation are thought to contribute to different extents to α-synuclein aggregation and fibrillation (Barrett and Timothy Greenamyre, 2015). Phosphorylation is the most relevant post-translational modification related to synucleinopathies. Increased levels of the phosphorylated protein at serine 87 have been found in human brains from LB dementia patients (Paleologou et al., 2010) and at serine 129 in LB from PD patients (Fujiwara et al., 2002). Although this latter has been widely investigated, its role on the pathogenesis of PD has not been determined yet. Some authors propose that it is implicated in α-synuclein pathological aggregation (Fujiwara et al., 2002) whereas others suggest that it has a protective role on the aggregation and toxicity (Oueslati et al., 2010; Chen et al., 2019). The protein also suffers C-terminal truncation linked to an increase of α-synuclein aggregation *in vitro* (Murray et al., 2003); the loss of SN neurons in transgenic mice overexpressing this form has been reported (Wakamatsu et al., 2008). Both the N- and C-terminal domains have also been involved in the interaction with proteins and metal ions (Miotto et al., 2014; Carboni and Lingor, 2015; Billings et al., 2016).

From a conformational point of view, under physiological conditions α-synuclein is considered to be an intrinsically unstructured protein. The soluble α-synuclein in the cytosol is natively unfolded (Burré et al., 2013). Conformational flexibility is largely attributed to the α-synuclein structure because it can adopt a range of conformations depending on its interactions with membranes or proteins (Lashuel et al., 2013). When α-synuclein/PL interaction occurs, the N-terminal domain takes on an α-helical configuration that occurs physiologically in a dynamic equilibrium with the soluble state (Eliezer et al., 2001). An α-helical homo-tetramer of the protein has recently been identified in neurons and other cells (Bartels et al., 2011; Wang et al., 2011; Fanning et al., 2018), occurring in equilibrium with α-synuclein monomers. As the tetramer is resistant to pathological aggregation, the promotion of its formation could be protective against α-synuclein oligomerization (Bartels et al., 2011; Dettmer et al., 2013, 2015a,b, 2017). Moreover, under pathological conditions, the induction of α-synuclein aggregation results in the formation of an initial population of oligomers enriched in insoluble cross-β-sheets (Conway et al., 2000; Pineda and Burré, 2017). These species may act as nuclei for the next steps of elongation and assembly into fibrils, which are finally deposited in LB (Wood et al., 1999; Lashuel et al., 2013). Another proposed mechanism for the formation of fibrillar aggregates is the lateral association of the oligomeric granules without nuclei formation (Bhak et al., 2009; Makwana and Sundd, 2016). Oxidative stress, post-translational modifications, accumulation of α-synuclein and changes in the levels of metal ions, PL, and fatty acids have been proposed as modulators of the aggregation process (Ruipérez et al., 2010; Lashuel et al., 2013; Galvagnion, 2017).

This review focuses on the intriguing characteristic of α-synuclein to pleiotropically interact with and modulate a variety of lipids and on how these interactions participate in the protein’s biological and pathological functions.

## Functions of α-Synuclein

The physiological cellular functions of α-synuclein are still a matter of intense debate, despite continuous efforts over the last 30 years to clarify the issue. Since it is highly concentrated in presynaptic terminals, where it is associated with synaptic vesicles (Maroteaux et al., 1988), the involvement of α-synuclein in neurotransmission, and synaptic plasticity has been extensively investigated (Burré, 2015; Zaltieri et al., 2015). Although there is disagreement as to whether it promotes or inhibits neurotransmitter release, it is well-established that α-synuclein mediates this process by regulating the availability of synaptic vesicles in different pools, facilitating their clustering, recycling, and docking to the cell membrane (Chandra et al., 2005; Burré et al., 2010; Zaltieri et al., 2015; Miraglia et al., 2018). The involvement of α-synuclein in synaptic vesicle endocytosis has been demonstrated (Vargas et al., 2014). In addition, α-synuclein can act as a chaperone molecule thus contributing to the assembly of the SNARE complex (Bonini and Giasson, 2005; Diao et al., 2013; Burré et al., 2014). The modulation of dopamine levels by α-synuclein has been demonstrated as a consequence of the inhibition of the neurotransmitter synthesis through the regulation of tyrosine hydroxylase activity (Abeliovich et al., 2000; Peng, 2005) and the inhibition of the vesicular monoamine transporter-2 (Guo et al., 2008). It also has a regulatory effect on the targeting and activity of the dopamine transporter DAT (Swant et al., 2011; Oaks et al., 2013; Butler et al., 2015).

α-Synuclein also has roles which are unrelated to synaptic functions. Surguchev and Surguchov have reviewed recent findings focused on its implication in the regulation of gene expression (Surguchev and Surguchov, 2017). A plethora of other biological functions have been attributed to α-synuclein such as regulation of apoptosis, modulation of glucose and calmodulin levels, and neuronal differentiation (Emamzadeh, 2016). Even though these latter functions have not been explored in depth, they provide evidence for proposing α-synuclein as a pleiotropic molecule.

β- and γ-synucleins have not been linked to the pathogenesis of PD. However, several reports have shown that β-synuclein has an inhibitory effect on the aggregation of α-synuclein (Brown et al., 2016) suggesting a neuroprotective role against synucleinopathies. Otherwise, it has been proposed that γ-synuclein is involved in cancer progression and metastasis (Hibi et al., 2009; Hua et al., 2009; Dunn et al., 2015).

## α-Synuclein and Membrane Lipids

Although the native α-synuclein three-dimensional conformation is still under discussion, differential folding states for physiological and pathological conditions have been assigned. One of the cutting-edge questions is the role of membrane lipids in α-synuclein conformation since protein-membrane binding has been associated with both normal and pathological functions. It has been reported that the α-synuclein/membrane interaction induces different α-helix states that could participate in protein function or drive aggregation (Bodner et al., 2010; Dikiy and Eliezer, 2012; Fares et al., 2014; Ysselstein et al., 2015; Burré et al., 2018; O’Leary et al., 2018). At the same time, the targeting of α-synuclein in its different conformations at the membrane surface can alter lipid composition, thus promoting pathological effects (van Rooijen et al., 2009; Reynolds et al., 2011; Tosatto et al., 2012; Hellstrand et al., 2013). A significant body of experimental evidence reinforces the hypothesis that oligomers, the initial state of protein aggregation, are the most neurotoxic species because of their ability, after interaction, to disrupt biological membranes (Danzer et al., 2007; Winner et al., 2011; Fusco et al., 2017; Galvagnion, 2017).

The nature of the interactions between α-synuclein and PLs has been extensively reviewed in several papers that mainly address this topic through biophysical studies (Dikiy and Eliezer, 2012; Pineda and Burré, 2017; Burré et al., 2018; O’Leary and Lee, 2018). Here we will discuss the most biologically relevant findings. One of the points included in this section is how specific PLs participate in the induction of conformational changes of α-synuclein and how this affects its biological and/or pathological functions.

After adipose tissue, the brain is the organ with the highest lipid content. PL content in the brain is approximately 6% of dry weight and is represented by two main classes of molecules: glycerophospholipids and sphingolipids (Lahiri and Futerman, 2007). A glycerophospholipid molecule consists of two fatty acids, one saturated and one unsaturated, esterified in sn-1 and sn-2 of the glycerol backbone, respectively. The brain is particularly enriched in two PUFA: arachidonic acid (AA, 20:4-ω-6) and docosahexaenoic acid (DHA, 22:6-ω-3) (Chen et al., 2008). PLs are the main constituents of cellular membranes and because of their amphipathic nature, they provide the necessary biophysical environment for ensuring the proper functioning of structural proteins, receptors, enzymes, and ion channels located at the cell surface or in intracellular membranes.

The plasma and organelle membranes are asymmetric; this characteristic determines specific and differential lipid composition in the inner and the outer leaflets. PC, PE, sphingomyelin, and Chol are the most abundant lipids in the outer hemimembranes. PS and PI are acidic PLs and are predominantly located in the inner hemimembranes. This particular lipid composition is determinant for the formation of lipid rafts, specific membrane liquid-ordered microdomains that govern protein recruitment (van Meer et al., 2008; Marquardt et al., 2015).

In native conditions, α-synuclein can be mainly found in two states: a soluble unfolded monomeric form or a membrane-associated multimeric form. The pathological state of the protein is composed of predominantly β-sheet oligomers and amyloid fibrils (Burré, 2015). PLs have been reported to participate in and modulate the different states of α-synuclein. Originally, α-synuclein was described as a monomer in equilibrium between the cytosol and the membrane fraction (i.e., plasma membrane or vesicles). The occurrence of these two physiological states has generated some controversy, but consensus has now been reached that α-synuclein mainly occurs as an intrinsically disordered monomer (Burré et al., 2013). Very recently, a helically folded tetramer of the soluble protein has been described in equilibrium with the monomer (Bartels et al., 2011; Wang et al., 2011; Fanning et al., 2018). Soluble α-synuclein exists as a random coil three dimensional structure that increases α-helical conformation after interacting with membrane PLs, suggesting that protein/PL interaction triggers structural changes that could modulate biological function (Davidson et al., 1998; Zhu et al., 2003). Furthermore, biophysical studies using tryptophan- and spin-labeled determinations demonstrated that the portion of the protein that interacts with the membrane adopts an α-helix conformation which is entirely buried within the depth of the membrane whereas the rest of the segments present lower membrane penetration and higher flexibility (Bussell, 2005; Wietek et al., 2013).

The preferred PLs for α-synuclein membrane binding are those with an acidic head group such as PS and PI (Shvadchak et al., 2011). The charged nature of these PLs makes lysine residues candidates for α-synuclein binding sites (Jo et al., 2000; Perrin et al., 2000). The interaction between the N-terminal of α-synuclein and PS seems to be critical for C-terminal SNARE-dependent vesicle docking (Lou et al., 2017). Since SNARE-dependent vesicle docking is necessary for calcium-mediated neurotransmitter release, it is reasonable to assume that α-synuclein/PS interaction is critical to the neurotransmission process at the synapse. Indeed, as previously mentioned, one of the best characterized biological functions of α-synuclein is its participation in vesicle recycling (Scott and Roy, 2012; Wang et al., 2014). Experiments performed in giant unilamellar vesicles composed of different proportions of dioleoyl-PC and several anionic PLs demonstrated that wild-type α-synuclein binding is dependent on the presence of negatively charged head groups. Binding to anionic PLs is also dependent of the liquid-ordered state of the vesicles, thus indicating that protein/membrane interactions are governed not only by electrostatic forces but also by lipid packaging and hydrophobic forces (O’Leary and Lee, 2018). The interaction of β- and γ-synucleins with membranes depends on the presence of PL acidic head group and is also determined by the curvature of the binding surface (Ducas and Rhoades, 2012).

A fact that argues in favor of the role of α-synuclein/lipid interaction in the etiopathogenesis of synucleinopathies is that all missense mutations responsible for familial PD are localized in the 11-residue repeat domain that has membrane-binding properties; indeed, these mutations alter the lipid binding properties (Jo et al., 2002; Fares et al., 2014; Ghosh et al., 2014; Robotta et al., 2017). A30P and E46K mutants display altered binding to anionic PLs, thus suggesting that membrane interaction could be disrupted in the familial forms of PD (Stöckl et al., 2008).

Binding to PS has been shown to induce an increase in α-synuclein oligomerization, thus providing evidence of the role of protein/PL interaction in the modulation of the pathological aggregation of the protein (Hu et al., 2016). After interacting with PLs, the impairing effects of α-synuclein on biological membranes could be explained by the increase in membrane tension and lipid extraction that promote pore formation and membrane lysis (Jo et al., 2000; Zhu et al., 2003; Tosatto et al., 2012; Braun and Sachs, 2015; Pan et al., 2018). In line with this, it has been proposed that the interaction between acidic PLs and α-synuclein plays a role during nuclear extrusion in erythroblast differentiation (Araki et al., 2018).

Phosphatidic acid (PA), another anionic PL, differs from PS, and PI mainly in terms of the small size of its head group; this particular characteristic allows its localization very close to the hydrophobic core of the lipid bilayer. PA is also an α-synuclein partner for membrane targeting (Perrin et al., 2000). Specifically, PA esterified with saturated/monounsaturated fatty acids is the preferred molecular species for α-synuclein binding and also induces an enhancement in protein aggregation through the induction of changes in the secondary structure (Mizuno et al., 2017). It has been reported that α-synuclein overexpression is also able to modulate enzyme pathways that produce PA (Dikiy and Eliezer, 2012) (see the section “α-Synuclein and Lipid Signaling”).

α-Synuclein has also been shown to be associated with mitochondrial membranes (Ghio et al., 2016). Binding of α-synuclein to mitochondrial membranes has been detected in SN dopaminergic neurons from mice and human brain (Li et al., 2007; Devi et al., 2008). The nature of protein association with mitochondria was determined by using liquid-disordered membranes enriched in cardiolipin, an essential PL very abundant in the inner mitochondrial membrane and also essential for organelle function. Cardiolipin-mediated binding of α-synuclein could represent a mechanism by which the cytosolic protein is targeted to mitochondria (Ghio et al., 2016). Once attached to the membrane surface, the protein is also able to extract lipids, which could explain the loss of mitochondrial integrity during dopaminergic neuronal degeneration (Pozo Devoto and Falzone, 2017).

The structural changes that α-synuclein undergoes after interacting with PLs in membranes and how these changes contribute to amyloidogenesis is nevertheless still under debate. There is agreement that acidic PLs are the preferred lipids for membrane targeting and that the protein/lipid ratio could be determinant for both biological function and amyloidogenic propensity. Very recently, it has been shown that the conformational state of α-synuclein depends on the protein/lipid ratio. A high α-synuclein/lipid ratio would promote protein/protein interaction and in consequence increase the propensity to form oligomers enriched in β-sheet conformation which in turn could also fibrillate and induce amyloid aggregation (Powers et al., 2017; Terakawa et al., 2018). A low α-synuclein/lipid ratio, on the other hand, would establish a condition of protein dilution, thus blocking amyloid fibrillation, and indeed maintaining physiological interactions with the membrane (Terakawa et al., 2018). Previously mentioned evidence describes the interaction of α-synuclein with membranes in the absence of post-translational modifications; however, it has been shown that post-translational modifications such as phosphorylated or acetylated forms of the protein would interact in a different way (Burré et al., 2018). One of the physiologically relevant post-translational modifications of α-synuclein is its acetylation in the N-terminal, which enhances binding to PC, a zwitterionic PL presented in micelles and small unilamellar vesicles with high curvature (r∼16–20 nm). Chol, another important membrane constituent, has been shown to reduce N-acetylated α-synuclein-PC binding. These *in vitro* results reported by O’Leary and coworkers suggest that N-acetylation promotes the binding of α-synuclein to highly curved membranes and that Chol interferes with this interaction (O’Leary et al., 2018). Moreover, N-acetylated α-synuclein has been shown to diminish the aggregation propensity triggered by ganglioside GM1 binding (Bartels et al., 2014). Phosphorylated α-synuclein in serine 129 has been studied in depth in the last decade, with disparate data being reported in relation to membrane interaction. However, the most accepted theory to emerge from *in vitro* and *in vivo* experiments is that the phosphorylated form has an inhibitory effect on α-synuclein association with the membrane (Fiske et al., 2011; Kuwahara et al., 2012; Nübling et al., 2014; Oueslati, 2016).

Phospholipids also participate in the spreading of α-synuclein, mainly attributed to exosomes, and are thus involved in synucleinopathy propagation. Mass spectrometry studies show the presence of PC, PE, PI, PS, and the gangliosides GM2 and GM3 in exosomes isolated from neuroblastoma cells (Marie et al., 2015). Moreover, experiments carried out on small unilamellar vesicles to ascertain the role of gangliosides in α-synuclein conformation demonstrated that GM1 and GM3, as well as exosomes, are able to accelerate the aggregation of the protein (Marie et al., 2015).

**FIGURE 1:**
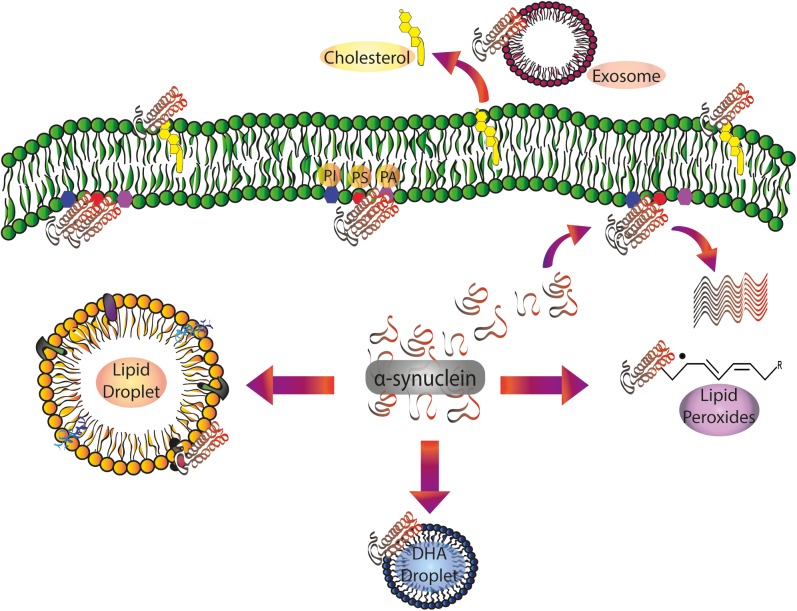
α-Synuclein/lipid interactions: from the plasma membrane to intracellular compartments and extracellular vesicles. Soluble α-synuclein exists as a random coil three-dimensional structure. Membrane targeting with acidic PLs (PS and PI) triggers α-helical folding at the N-terminal domain. Protein pathological conformation is predominantly represented by β-sheet oligomers and amyloid fibrils. α-Synuclein pathological aggregation is dependent on the protein/lipid ratio. Spreading of α-synuclein, largely attributed to exosomes, is also regulated by its interaction with the PLs of the extracellular vesicles. Free fatty acids, mainly DHA, are able to induce α-synuclein oligomerization. After the interaction with lipid peroxidation products, α-synuclein is prone to the formation of toxic stable oligomers. α-Synuclein also regulates Chol efflux via ABCA1 transporter. DHA, docosahexaenoic acid; PA: phosphatidic acid; PI, phosphatidylinositol; PS, phosphatidylserine.

Despite the mounting evidence mentioned above, the question of how PL interaction intervenes in α-synuclein physiological and pathological function remains unanswered (Figure 1). In order to shed further light on the specific role of lipid interactions in α-synuclein biology and pathology and how these interactions occur *in vivo*, it is necessary to carry out additional experiments using models that mimic living cell lipid composition, membrane asymmetry, and the intracellular environment.

## α-Synuclein and Lipid Droplets

Neutral lipids are part of a large, heterogeneous family of compounds with the distinctive characteristic of being hydrophobic molecules. Another of their particular features is the lack of charged groups. The two main classes of neutral lipids in mammals are TAGs and Chol esters, both of which reside in the cytosol, contained in a simple membrane-surrounded organelle, the LD (Welte, 2015). LD membranes contain PLs, unesterified Chol, and specific anchored proteins with signaling and lipolytic functions (Gao et al., 2017; Sztalryd and Brasaemle, 2017); as highly dynamic storage organelles, they provide a rapidly mobilizable TAG pool to act as an energy source or as a fatty acid donor for lipid remodeling processes.

Though LDs are present in almost all cell types, their major occurrence is in adipocytes and hepatocytes. They also serve as cellular stress indicators and are associated with pathological states including starvation, infection, cancer, and liver steatosis (Gluchowski et al., 2017; Tirinato et al., 2017). Scarce information on the precise function of LDs on the nervous system is available in the literature. However, recent works correlate the appearance of LDs with pathological situations in neurodegenerative disorders. Glial cells were reported to be LD-positive cells that accumulate neutral lipids in response to the injury of neighbor neurons in several neurodegenerative conditions (Liu et al., 2015; Cabirol-Pol et al., 2018). LDs were detected in Aplysia axons and neuronal primary cultures and neurons of Huntington’s disease models (Savage et al., 1987; Martinez-Vicente et al., 2010; Welte, 2015). In addition, iron accumulation, an event intensively described in several neurodegenerative processes and also in PD, has been shown to increase LD formation in dopaminergic neurons (Salvador, 2010; Schneider and Bhatia, 2012; Sánchez Campos et al., 2015).

A large body of evidence points to α-synuclein/LD interaction. Experiments performed *in vitro* demonstrate that α-synuclein binds to artificial 60 nm diameter LDs (Thiam et al., 2013). Moreover, it has been shown that in HeLa cells incubated with a high concentration of fatty acid, wild-type α-synuclein translocates from the cytosol to the surface of LDs (Colebc et al., 2002). α-Synuclein mutants display a different behavior: whereas A53T strongly associates with LDs, A30P remains cytosolic. These findings were corroborated by the heterologous expression of human α-synuclein in *Saccharomyces cerevisiae* (Sere et al., 2010). The biological significance of the α-synuclein-induced increase in LDs observed in yeasts was addressed in a mutant strain unable to synthesize neutral lipids and more tolerant to α-synuclein overexpression (Sere et al., 2010). In line with these findings, we recently reported that A53T α-synuclein overexpression in dopaminergic neurons induces an increase in TAG production and LD accumulation (Sánchez Campos et al., 2018). Fe exposure in A53T α-synuclein-overexpressing neurons increases protein aggregation, thus also augmenting TAG and LD content (Sánchez Campos et al., 2018). The nature of the α-synuclein/LD interaction has been shown to depend on PL packing at the LD membrane, being not fully packed monolayers the preferred surface for protein binding (Thiam et al., 2013). LD surface has been described as a higher hydrophobic environment than the PL bilayer (Thiam et al., 2013; Kory et al., 2016). The need for this highly hydrophobic organelle has been reported in other biological processes such as protein viral assembly (Ogawa et al., 2009; Welte and Gould, 2017).

The relevance of the biological interaction between α-synuclein/LD has not been explored *in vivo*, but it can be proposed that LDs are early markers of neurodegenerative processes and the characteristic of these vesicles make them good candidates for the modulation of α-synuclein conformational changes and pathological aggregation. Further research is required for a deeper understanding of these phenomena and their implications in synucleinopathies (Figure 1).

## α-Synuclein and Fatty Acids

Another interesting discussion is the role of FFA in α-synuclein biology. Brain membranes exclusively contain a high concentration of PUFA, DHA, and AA being the most abundant. DHA is an essential fatty acid that needs to be incorporated from the diet, and disturbances in its metabolism have been reported to be associated with both neurodevelopment and neurodegenerative disorders (Chen et al., 2008). Some years ago, several *in vitro* observations proposed that α-synuclein could act as a fatty acid binding protein in the brain (Sharon et al., 2001). Soon afterward, PUFA were shown to promote the assembly of α-synuclein soluble oligomers (Sharon et al., 2003a; Karube et al., 2008). Both DHA and to a lesser extent AA are able to induce α-synuclein oligomerization as demonstrated by transmission electron microscopy, electrophoresis of native gels, and fluorescence assays with thioflavin T (Broersen et al., 2006). Relative concentrations of α-synuclein and DHA appear to govern the free and bound states of the protein and also its conformational changes, by increasing its α-helix structure (Sharon et al., 2001; Fecchio et al., 2018). Moreover, prolonged exposure to PUFA triggers the formation of α-synuclein fibrils. The establishment of oligomeric or fibrillar conformations in the presence of DHA depends on the protein/FFA ratio. Whereas fibril formation is favored by a 1:10 molar ratio, oligomeric species characterized by lack of seeding properties for fibrillation are stabilized by a 1:50 ratio (De Franceschi et al., 2011). The general consensus among the broad range of studies on the effect of fatty acids on α-synuclein biology is that PUFA elicit the formation of diverse oligomeric forms with differential structural and biological properties (Perrin et al., 2001; Fecchio et al., 2013). Oligomeric species generated *in vitro* by DHA exposure showed a partial α-helical structure, with the capacity to alter the membrane permeability and thus to trigger cytotoxicity (Fecchio et al., 2013). β- and γ-synucleins are also able to interact with PUFA trough their lipid binding domain (Perrin et al., 2001).

A rise in free DHA levels in cytosolic fractions has already been found in brains of patients with PD and LB dementia, and this could be responsible for the oligomerization of α-synuclein and the underlying neuronal damage (Sharon et al., 2003b). Biophysical studies combined with transmission electron microscopy demonstrated that DHA in solution is able to form oil droplets. These oil droplets can be remodeled by α-synuclein, reducing their size and fatty acid concentration (Broersen et al., 2006; De Franceschi et al., 2009; Figure 1). In addition, the interaction with DHA droplets promotes differential susceptibility of proteolytic degradation (De Franceschi et al., 2009).

α-Synuclein has been shown not only to form covalent bonds with DHA but also to act as a scavenger of the oxidation products of DHA, mainly through its histidine residue in position 50 (De Franceschi et al., 2017). Moreover, it has been observed that lysine residues, acting as nucleophiles, are able to interact with electrophilic products of lipid peroxidation and that this protective effect is weakened after lysine acetylation. Additionally, α-synuclein binding to liposomes containing PC and PA enriched with PUFA has been reported to have a protective effect against nitration and oxidation (Trostchansky et al., 2006). The interaction of α-synuclein with 4-hydroxy-2-nonenal, one of the main products of PUFA peroxidation, is able to promote the formation of toxic stable oligomers, and also prevent the passage toward the final state of fibrils (Qin et al., 2007; Puspita et al., 2017; Shamoto-Nagai et al., 2018). Furthermore, dopaminergic cells incubated with free PUFA showed decreased viability associated with the presence of oligomeric α-synuclein (Assayag et al., 2007).

The reported findings highlight the connection between oligomeric α-synuclein and fatty acid oxidation in dopaminergic toxicity, suggesting the existence of a positive loop of increased damage when α-synuclein is allowed to interact with PUFA and their oxidation products. One of the most accepted theories for the onset of neurodegenerative diseases associated with aging postulates the increase in oxidative stress as one of the main gating factors. Since the well-described decrease in antioxidant defenses associated with the aging process increases oxidative stress, it is arguable that lipid peroxides could be available for the interaction with α-synuclein thus triggering the generation of toxic oligomeric forms. Moreover, the extensively documented decrease in brain PUFA content during aging could also be responsible for an increase in saturated PLs at the membranes that in turn could decrease the association with α-synuclein and augment the cytosolic levels of the protein available for binding with lipid peroxides (López et al., 1995; Giusto et al., 2002; Mateos et al., 2010; Ledesma et al., 2012). Studies on oligodendroglial cells overexpressing the A53T mutant have shown that oxidative stress exposure after supplementation with DHA is able to promote the genesis of inclusion bodies whose composition includes phosphorylated α-synuclein (serine 129) and ubiquitinated and SUMOylated proteins, all reported to be aggregation inducers (Riedel et al., 2011).

A number of PD animal models have been used to corroborate some of the above-discussed *in vitro* results. The use of transgenic mice overexpressing different α-synuclein forms and subjected to high-DHA diet has served to support some of the earlier findings. Yakunin et al. (2012) showed increased accumulation of soluble and insoluble α-synuclein forms accompanied by astrocytosis and neuritic defects in an A53T transgenic mouse model fed a DHA-enriched diet. Experiments *in vivo* with transgenic mice expressing the A53T α-synuclein mutant demonstrated that a diet with high DHA content reduces lipoxidative damage (Muntané et al., 2010). A very recent report demonstrated that a diet supplemented with n-3 PUFA promotes the accumulation of a 42-kDa oligomeric form of the protein (Yakunin et al., 2012; Coulombe et al., 2018). Analysis of *post-mortem* human brains corroborates some of the previous *in vitro* and *in vivo* experimental findings. Protein profile of synucleinopathy patients was enriched with both soluble and insoluble oligomers and a positive correlation between the accumulation of α-synuclein oligomers and cytosolic DHA content was reported (Sharon et al., 2003a,b).

All these findings postulate DHA in soluble and esterified forms as an important candidate for modulating both the targeting and the biological function of α-synuclein. Thus, the regulation of DHA availability under pathological conditions could be an important event for regulating α-synuclein oligomerization and aggregation processes. In this sense, lipid pathways involved in DHA acylation, and deacylation could constitute a promising strategy for new therapeutic approaches in synucleinopathies (see section “α-Synuclein and Lipid Signaling”) (Figure 1, 3).

## α-Synuclein and Lipid Metabolism

**FIGURE 2:**
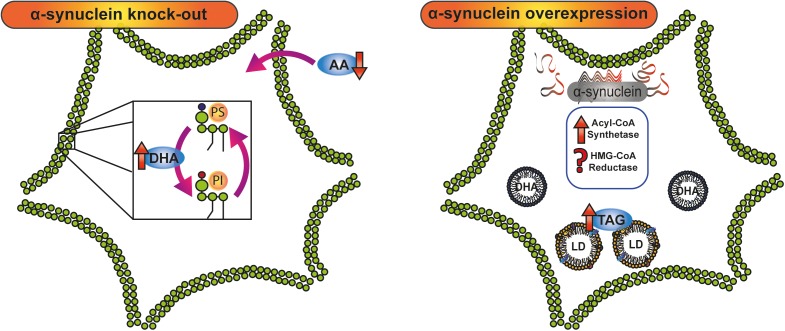
Crosstalk between α-synuclein and lipid metabolism. Knock-down and overexpression of α-synuclein have demonstrated the involvement of the protein in lipid metabolism. In α-synuclein knock-out mice, AA and DHA, the main polyunsaturated fatty acids in the brain, are oppositely metabolized: whereas AA intake is diminished, DHA turnover and acylation in PI and PS are increased. α-Synuclein overexpression triggers an increase in TAG content and LD accumulation by stimulation of acyl-CoA synthetase activity and probably the modulation of HMG-CoA reductase. AA, arachidonic acid; DHA, docosahexaenoic acid; HMG-CoA, 3-Hydroxy-3-methyl glutaryl coenzyme A; LD, lipid droplet; PI, phosphatidylinositol; PS, phosphatidylserine; TAG, triacylglycerol.

The clear involvement of a crosstalk between α-synuclein and lipid metabolism was investigated by Murphy’s group using protein knock-out mice (Castagnet et al., 2005; Golovko et al., 2006, 2007, 2009; Barceló-Coblijn et al., 2007; Golovko and Murphy, 2008). SNCA (-/-) mice oppositely metabolize the two main brain PUFA, AA, and DHA. The lower intake of AA reported both in neurons and astrocytes from SNCA (-/-) mice was reverted by exogenous wild-type α-synuclein through the modulation of acyl-CoA synthetase activity (Golovko et al., 2006). However, DHA turnover and acylation in PE, PI, and PS was found to be increased in SNCA (-/-) mice when compared with SNCA (+/+) mice (Golovko et al., 2007). This differential effect in AA and DHA metabolism as a consequence of the silencing of α-synuclein argues in favor of compensatory mechanisms associated with PUFA content in brain membranes (Figure 2).

Several experimental evidences have demonstrated that both the absence and the overexpression of α-synuclein are able to disturb lipid homeostasis by increasing neutral lipid storage. In astrocytes and whole brain, the absence of α-synuclein switches the distribution of fatty acids to neutral lipid reserves and also increases neutral lipid mass (Castagnet et al., 2005; Barceló-Coblijn et al., 2007). Surprisingly, α-synuclein overexpression gives rise to abnormal lipid metabolism in yeasts, characterized by an increase in LD content (Outeiro and Lindquist, 2003; Su et al., 2010). In line with this, overexpression of wild-type and mutant forms of α-synuclein triggers the accumulation of LDs in HeLa cells, and in human-induced pluripotent stem cell (Colebc et al., 2002; Fanning et al., 2018). Regarding these findings, it can be suggested that physiological levels of α-synuclein are necessary for lipid homeostasis. γ-Synuclein, another member of the synuclein family, has also been implicated in the maintenance of LD formation and in the modulation of lipid composition in adipocytes and the nervous system in mice (Guschina et al., 2011; Voshol et al., 2012).

In harmony with these results, a recent study in our laboratory showed that the overexpression of A53T α-synuclein induces an increase in TAG content and the accumulation of LDs in N27 dopaminergic neurons (Sánchez Campos et al., 2018). The rise in neuronal TAG content was associated with increased fatty acid synthase expression and Acyl-CoA synthetase activity, with no variations in TAG lipase activity or in fatty acid β-oxidation, thus demonstrating that the overexpression of A53T α-synuclein triggers a lipid metabolic switch in dopaminergic neurons. Pharmacological blockage of TAG *de novo* synthesis renders the neurons more susceptible to iron-induced oxidative stress (Sánchez Campos et al., 2018).

α-Synuclein biology and Chol metabolism have been shown to interact in many different ways. Such intersections regulate one another in a complex and not yet fully understood manner (Yeger-Lotem et al., 2009; Galvagnion, 2017). Controversial information coexists regarding plasma Chol level since it has been considered as either positive or negative risk factor for PD, or even not being linked at all to PD (Galvagnion, 2017). More concrete evidence links oxidized Chol derivatives with the onset and progression of PD (Bosco et al., 2006; Marwarha and Ghribi, 2015). In this regard, 27-hydroxycholesterol, a product of Chol oxidation, has been found to be increased in the brain, cortex and plasma, of PD patients (Lee et al., 2009; Seet et al., 2010; Cheng et al., 2011). Moreover, exogenous 27-hydroxycholesterol is able to induce the expression and accumulation of α-synuclein in human dopaminergic neurons through a transcriptional mechanism mediated by the transcription factor LXRβ (Marwarha et al., 2011). A possible mechanism that supports these findings could be the regulation of Chol efflux from neuronal cultures via ABCA1 transporter associated with the increased levels of oxidative stress reported in PD (Hsiao et al., 2017).

Regarding Chol biosynthesis, *in silico* analysis from the Yeger-Lotem laboratory identified the ergosterol-mevalonate pathway as a candidate for the cellular response to α-synuclein toxicity. One of the identified genes is Hrd1, a ubiquitin protein ligase related with the regulation of HMG-CoA reductase, the rate-limiting enzyme for the *de novo* Chol synthesis (Yeger-Lotem et al., 2009). They also found that pharmacological inhibition of HMG-CoA reductase by statins makes yeasts more vulnerable to α-synuclein toxicity (Yeger-Lotem et al., 2009).

Our results in dopaminergic neurons and those obtained in yeasts suggest a protective role of neutral lipids (TAG and Chol) during α-synuclein overexpression either against proteotoxicity or against oxidative stress (Yeger-Lotem et al., 2009; Auluck et al., 2010; Sánchez Campos et al., 2018). However, contrasting effects have been reported in primary human neurons treated with statins (Bar-On et al., 2008; Roy and Pahan, 2011). In view of the above, additional studies are needed to understand the biological significance of these controversial experimental evidences. Concerning this matter, the clinical relevance of statins is still under debate and is currently being addressed in a trial with PD patients treated with simvastatin which ends in 2020 (Carroll and Wyse, 2017).

The accumulated data allows us to propose a biological function for α-synuclein as a lipid metabolism modulator. Both at physiological levels and when overexpressed, α-synuclein is able to trigger a metabolic switch involving several aspects of lipid biology (Figure 2). The main biological response to α-synuclein overexpression is an increase in neutral lipid content that could be related to a neuroprotective strategy, whereas under physiological conditions the modulation of AA levels by the protein appears to be associated with prostaglandin generation and thus with a role in the inflammatory cascade (Golovko et al., 2007).

The open question is how α-synuclein triggers the above-mentioned metabolic changes. One possibility is that the switch in neutral lipid metabolism in terms of LD accumulation could be a consequence of endoplasmic reticulum stress and autophagy impairment, triggered by proteotoxicity due to the overexpression of α-synuclein (Velázquez et al., 2016; Garcia et al., 2018; Pitcairn et al., 2018). The other possibility is that α-synuclein could be involved in the regulation of gene expression associated with lipid metabolism. This latter possibility relates to the high DNA affinity of amyloidogenic proteins, the nuclear localization of α-synuclein, and its participation in epigenetic modifications through histone acetylation (Ma et al., 2014; Surguchev and Surguchov, 2017). Specifically, it has been described that α-synuclein modulates gene expression related with cell survival by decreasing H3 acetylation (Pavlou et al., 2017) and with ubiquitination (Martins et al., 2011). Additional studies will be required to ascertain which of the above-mentioned mechanisms are biologically and pathologically involved and their link with lipid metabolism.

## α-Synuclein and Lipid Signaling

Apart from their structural and biophysical role in biological membranes, lipids also serve as reservoirs of intracellular and extracellular messengers. One of the most investigated events in lipid signaling is the action of phospholipases and PL kinases and phosphatases that produce different lipid and non-lipid messengers from membrane PLs.

Phospholipases catalyze the hydrolysis of acyl and phosphate esters and are named according to their hydrolyzed position on the PL molecule. Among them, PLA2 and phospholipases C and D (PLC and PLD) are widely studied and many of them have been implicated in neurodegenerative processes.

Phospholipases A2 catalyzes the hydrolysis of the fatty acid esterified in the sn-2 of the glycerol backbone of PL moieties and releases FFA and a lysophospholipid. There are currently six different known types of PLA2: secreted (groups I, II, III, V, IX, X, XI, XII, XIII, and XIV), cytosolic (cPLA2, group IV), calcium-independent (iPLA2, group VI), lipoprotein (groups VII and VIII), adipose (group XVI) and lysosomal (group XV), together constituting an enzyme superfamily (Dennis et al., 2011; Vasquez et al., 2018). In terms of neurodegenerative processes, and specifically PD, the most studied PLA2 isotypes are cPLA2 and iPLA2. The classical role assigned to iPLA2 enzymes is their active participation in membrane homeostasis through PL remodeling in Lands cycle where phospholipase activity operates coordinately with PL-acyltransferases. Later findings postulate other important functions for iPLA2s: while lysophosphatidyl moieties have been shown to modulate store-operated calcium entry, the released fatty acid, mostly DHA, has been described as the preferred substrate for the production of resolvins, lipid mediators involved in the resolution of inflammatory processes (Bazan, 2005; Serhan and Petasis, 2011). iPLA2 are mainly encoded by the PLA2G6 gene. In the nervous system, iPLA2β has been shown to be essential for remodeling membrane PLs in axons and synapses. Mutations in PLA2G6 have been associated with several neurodegenerative disorders, including adult-onset dystonia-parkinsonism, PARK14. In this regard, PLA2G6 knock-out mice have been widely used for the study of neuronal membrane-associated degeneration (Sumi-Akamaru et al., 2016). Indeed, increased expression of α-synuclein has been associated with mitochondrial injury triggered by iPLA2 dysfunction in PLA2G6 knock-out animals (Sumi-Akamaru et al., 2016). Moreover, the impairment of iPLA2-dependent calcium signaling has recently been implicated in neuronal loss through autophagic dysfunction in dopaminergic neurons derived from fibroblasts from PD patients and PLA2G6 knock-out animals (Zhou et al., 2016).

cPLA2 is mainly associated with the release of AA, the obligate substrate for prostaglandins generation catalyzed by cyclooxygenase-2, which is considered one of the main pathways mediating inflammatory response. Though neuroinflammation is a hallmark of PD, the specific PLA2 isoforms that are activated in dopaminergic neuronal damage have not been fully described. Besides the classical inflammatory process, the exposure to extracellular α-synuclein is able to activate cPLA2 and to promote synapse damage induced by phospholipase-dependent activity (Bate and Williams, 2015).

Upon PLA2 activation, it is important that fatty acid release be properly coupled to PL acylation activity; impairment of the Lands cycle could be responsible for an increase in FFA availability (DHA, AA). This increase in FFA availability, could in turn, be associated with α-synuclein and promote its oligomerization. The rise in cytosolic/soluble DHA content reported by Sharon and coworkers in *post-mortem* brains in PD and LB dementia patients is in agreement with the activation of any specific PLA2 that could consecutively induce α-synuclein oligomerization and aggregation (Sharon et al., 2003a). The workings of this mechanism still lack clarity.

**FIGURE 3:**
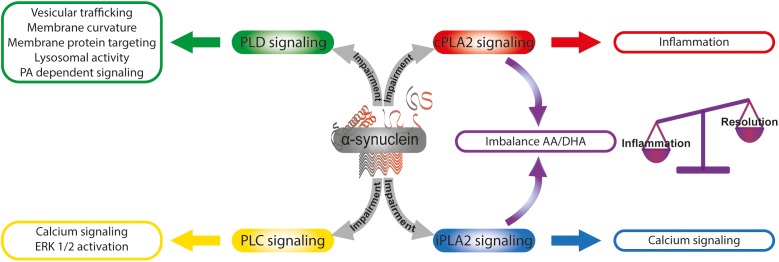
α-Synuclein and the modulation of lipid signaling. α-Synuclein is able to intervene lipid signaling by the modulation of phospholipase activities. The α-synuclein -induced impairment of cPLA2 and iPLA2 signaling triggers an imbalance in the AA/DHA ratio that finally could affect the balance of inflammation and resolution processes. Calcium signaling and ERK 1/2 activation are modulated by α-synuclein through PLC pathway impairment. α-Synuclein intervention in PLD signaling impacts on diverse cellular functions as vesicular trafficking, membrane curvature, and membrane protein targeting, lysosomal activity and PA-dependent signaling. AA, arachidonic acid; DHA, docosahexaenoic acid; PA, phosphatidic acid; cPLA2, calcium-sensitive cytosolic phospholipase A2; iPLA2, calcium-independent phospholipase A2; PLC, phospholipase C; PLD, phospholipase D.

The fact that SNCA knock-out mice present enhanced prostaglandin generation during neuronal injury argues in favor of a biological role for α-synuclein in the regulation of the inflammatory cascade (Golovko and Murphy, 2008). It has been demonstrated that DHA, mainly released from PLs by iPLA2 action, has a long half-life compared with other fatty acids and that its free form is the preferred substrate for neuroprotectin synthesis, and the resolution of the inflammatory process (Murphy, 2013). An important question is whether the dyshomeostasis between cPLA2 and iPLA2 triggered by α-synuclein overexpression could be responsible for a differential AA/DHA ratio and availability, thus modulating the balance between inflammation and resolution processes and promoting the neuroinflammatory phenotype in PD (Figure 3).

Among the classical signaling pathways associated with G-protein-coupled receptors is PI-specific PLC. Upon receptor activation, PLC hydrolyzes PI 4,5-bisphosphate to produce the lipid messenger diacylglycerol and the soluble inositol triphosphate. There are now six different families of PLC (β, γ, δ, 𝜀, ζ, and η) described in different tissues, with pleiotropic functions associated with the membrane receptor to which they are coupled (Kadamur and Ross, 2013). It has recently been shown that α-synuclein overexpression interferes with Gq/PLC-β signaling by preventing the rise in cytosolic calcium and ERK 1/2 activation (Volta et al., 2017). α-Synuclein is also able to trigger the impairment of G-protein signaling associated with sphingosine-1-phosphate by promoting the exclusion of the receptor from lipid rafts (Mohamed Badawy et al., 2018).

Phospholipase D signaling impairment has been reported in Alzheimer’s disease and the signaling pathway is, therefore, an emerging therapeutic target for this neurodegenerative disorder (Brown et al., 2017). The role of PLD in α-synuclein-associated pathologies, however, still lacks clarity. Classical PLDs, PLD1 and PLD2, catalyze the hydrolysis of the PC head group in order to produce PA. PA has pleiotropic functions both as a lipid messenger, regulating the activity of signaling proteins, and as an important modulator of membrane curvature. Whereas neuronal PLD1 has been associated with cytoskeleton architecture, PLD2 activity is linked to molecular events triggered by the activation of membrane receptors (Watanabe et al., 2011; Comoglio et al., 2014). Both PLD1 and PLD2 have been reported to participate in astroglial differentiation (Burkhardt et al., 2015). Genetic deletion of PLD1 or PLD2 in transgenic mice demonstrates that both enzymes participate in brain development and cognitive function (Burkhardt et al., 2014).

The role of classical PLDs in the several processes of the nervous system has been well-established, but their participation in neurodegenerative processes associated with synucleinopathies is not clear. *In vitro* and *in vivo* assays have given rise to a number of divergent reports of the role of α-synuclein in PLD signaling. *In vivo* experiments have demonstrated that α-synuclein-induced PLD2 inhibition is able to prevent dopaminergic neurodegeneration (Gorbatyuk et al., 2010), whereas *in vitro* assays using recombinant α-synuclein and purified PLD1 and PLD2 have shown no inhibition of PLD activity (Rappley et al., 2009). However, protein profile studies in *post-mortem* brains from PD patients display diminished PLD1 expression (Bae et al., 2014). In yeast, it has been demonstrated that PLD inhibition elicited by α-synuclein was related to dysregulation of lipid metabolism and trafficking (Outeiro and Lindquist, 2003). In line with this, our laboratory has recently described that the overexpression of human α-synuclein in neuroblastoma cells is able to induce a decrease in PLD1 mRNA and protein levels and to impair ERK1/2 signaling (Conde et al., 2018). In this cellular model of synucleinopathy, we demonstrated that the inhibition of PLD1 expression and the impairment of ERK1/2 signaling triggered by α-synuclein overexpression are associated with a decrease in neurofilament light chain expression and in consequence with alteration of the neuronal cytoskeleton. The reported findings highlight the biological significance of PLD regulation exerted by α-synuclein that could modulate PA levels in a compartment-specific manner, thus impacting on different cellular functions: (i) vesicular trafficking, (ii) membrane curvature and membrane protein targeting, (iii) lysosomal activity, and (iv) PA-dependent signaling, among others (Figure 3).

It is clear from the above that α-synuclein, mainly when it is overexpressed, is able to disturb lipid signaling. The plethora of lipid messengers (PA, AA, DHA, and diacylglycerol) able to be produced due to α-synuclein could have different impacts on important cellular processes in the nervous system such as vesicular trafficking, autophagy, cytoskeleton architecture, and neuroinflammation. Dissecting the signaling pathways responsible for the production of each lipid messenger and the specific cellular compartment where they work, could contribute to the development of new strategic therapies for synucleinopathies.

## Concluding Remarks

This review covers the diversity of ways in which α-synuclein interacts and connects with lipid biology. Much of the experimental evidences dealing with the physical interaction of the protein with different lipid moieties derive from biophysical studies performed on artificial membranes. It is clear that the α-synuclein N-terminal domain is able to interact with the majority of PLs and with Chol with different affinities. Further research is required in order to shed more light on precisely which of these α-synuclein/lipid interactions occur *in vivo*; clear findings will undoubtedly contribute to a fuller understanding of both the biological and pathological implications of α-synuclein.

Efforts toward a more detailed characterization of α-synuclein interventions in lipid metabolism and signaling will open the way to a more in-depth assessment of the protein’s implications for therapeutic purposes. One hypothesis to be tested is whether α-synuclein pleiotropic properties are able to trigger a lipid metabolic and signaling switch, creating a propensity to interact and aggregate thus establishing a positive feedback. Unraveling this paradigm could provide not only new insights into the biological role of α-synuclein but also innovative ways of devising strategies for the treatment of synucleinopathies.

## Author Contributions

GS contributed to conception and design of the study. GS, NA, and PIG organized the database and wrote the sections of the manuscript. MC designed and performed the figures. RU performed the critical reading and editing. All authors contributed to manuscript revision, read, and approved the submitted version.

## Conflict of Interest Statement

The authors declare that the research was conducted in the absence of any commercial or financial relationships that could be construed as a potential conflict of interest.

## Abbreviations

AA: arachidonic acid
Chol: cholesterol
DHA: docosahexaenoic acid
FFA: free fatty acids
HMG-CoA: 3-hydroxy-3-methylglutaryl coenzyme A
LB: Lewy bodies
LD: lipid droplet
PA: phosphatidic acid
PC: phosphatidylcholine
PD: Parkinson’s disease
PE: phosphatidylethanolamine
PI: phosphatidylinositol
PL: phospholipid
PLA2: phospholipases A2
PLC: phospholipase C
PLD: phospholipase D
PS: phosphatidylserine
PUFA: polyunsaturated fatty acids
SN: *substantia nigra pars compacta*
TAGs: triacylglycerols
